# Efficient *in vivo* generation of CAR T cells using a retargeted fourth-generation lentiviral vector

**DOI:** 10.1016/j.ymthe.2025.07.006

**Published:** 2025-07-16

**Authors:** Tiziana Coradin, Amy L. Keating, Alun R. Barnard, Lynsey Whilding, Diana Pombal, Zara Hannoun, Jack Lewis, Gayathri Devarajan, Sharifah Iqball, Emma Burton, Sara Ferluga, Daniel M. Jones, Ben M. Alberts, Jordan Wright, Daniel C. Farley, Deirdre M. O’Connor, Ravi M. Rao, Kyriacos A. Mitrophanous, Yatish Lad, Rachael Nimmo

**Affiliations:** 1 Oxford Biomedica (UK) Ltd., Oxford OX4 6LT, UK; 2 iosBio Ltd., Haywards Heath RH16 1DB, UK; 3 Department of Oncology, University of Oxford, Oxford OX3 7DQ, UK; 4 Mogrify Limited, Cambridge CB4 0FW, UK; 5 Gilead Sciences Ltd, 280 High Holborn, London WC1V 7EE, UK; 6 Chimeris UK Ltd., Cambridge CB22 3EE, UK; 7 Zelluna Immunotherapy, 0379 Oslo, Norway; 8 Oxford Nanopore Technologies, Oxford OX4 4DQ, UK; 9 Gene Therapy Vector Facility, Centre for Gene Therapy and Regenerative Medicine, King’s College London, London SE1 9RT, UK; 10 Barinthus Biotherapeutics plc, Harwell OX11 0DF, UK; 11 AviadoBio, London E14 5GX, UK; 12 Sitryx Therapeutics, Oxford OX4 4GA, UK; 13 Elexion Consulting Ltd, Abingdon, UK; 14 Nucleome Therapeutics Ltd., Oxford OX2 0HY, UK

**Keywords:** *in vivo* CAR T, lentiviral vectors, CAR T cells, immunotherapy, gene therapy

## Abstract

Chimeric antigen receptor (CAR) T cell therapy has proved remarkably successful for the treatment of hematological malignancies. However, the bespoke manufacturing of autologous CAR T cells is complex and expensive. The development of methods for *in vivo* engineering of T cells will enable generation of CAR T cells directly within the patient, bypassing the need for *ex vivo* manufacturing and thereby enabling greater access for patients. Here, we describe development of an improved retargeted Nipah envelope system paired with a fourth-generation lentiviral vector capable of specifically targeting T cells with increased efficiency, which generates high levels of functional CAR T cells *in vivo*. The retargeted vectors exhibited greater specificity to T cells compared to the VSV-G pseudotyped vector. Vectors targeted to either CD3 or CD8 similarly generated high levels of CAR T cells, which rapidly eradicated B cells, suggesting that T cell receptor (TCR) engagement is not required for lentiviral vectors to efficiently transduce T cells *in vivo*. Furthermore, the fourth-generation lentiviral vector platform (referred to as the TetraVecta system) employs the TRiP system to prevent incorporation of CAR protein into the vector particles, minimizing the risk of inadvertent transduction of tumor cells.

## Graphical abstract

**Figure:**
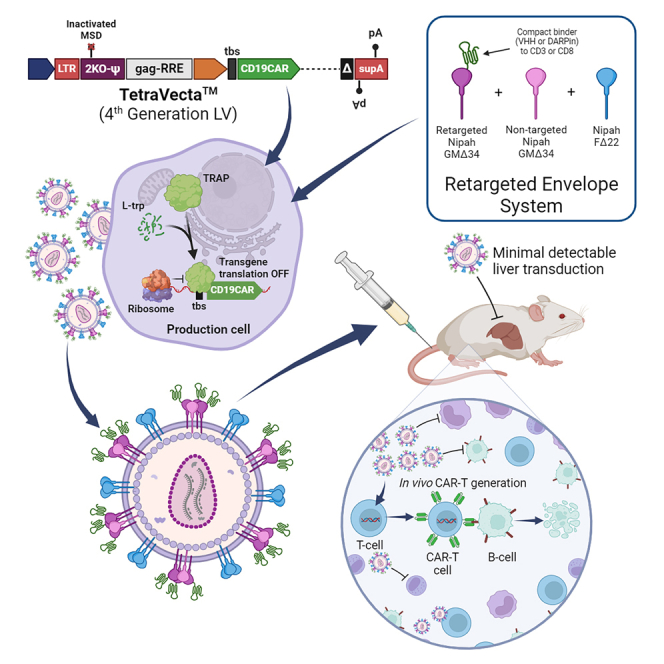

## Teaser

Nimmo and colleagues describe the development of an improved vector system for safe, specific, and efficient generation of CAR T cells *in vivo*, which will avoid the cost and complexity of *ex vivo* CAR T cell manufacturing and enable more patients to benefit from these highly effective therapies in future.

## Introduction

Chimeric antigen receptor (CAR) T cells have demonstrated remarkable efficacy in treating B cell malignancies. Seven different CAR T products have received marketing approval, including for earlier lines of therapy. However, access to these potentially curative therapies is restricted by the high cost of manufacture and the complex logistics of generating autologous cell therapies. Off-the-shelf approaches have been developed using allogeneic donor cells to alleviate some of these issues, but the long-term efficacy of these therapies is limited by the rejection of the donor cells.^1^ An alternative approach aims to bypass the requirement for *ex vivo* manufacturing by generating CAR T cells *in vivo*, directly within the patient.

A variety of methods have been developed for engineering of T cells *in vivo*, including viral and non-viral gene-transfer approaches.^2^ Lentiviral (LV) vectors are highly efficient at transducing activated T cells *in vitro* and have the desirable ability of achieving long-term expression of the CAR transgene in T cells through genomic integration. However, the pantropic vesicular stomatitis virus glycoprotein (VSV-G) used to pseudotype LV vectors for *ex vivo* T cell transduction is less suitable for *in vivo* engineering, where the vector should ideally be targeted to the specific cell type of interest. Two companies have recently entered into clinical trials to evaluate *in vivo* CAR T cell generation using targeted LV vectors pseudotyped with either a cocal envelope protein or CD7-targeted modified VSV-G envelope to deliver the CAR transgene to T cells.^3^^,^^4^ Additional proteins have been included with the cocal envelope to engage with CD3 and co-stimulatory receptors with a view to enhancing transduction of T cells.

Paramyxoviral envelopes have proved particularly useful for generating targeted LV vectors due to the separate attachment and fusion proteins, which allows retargeting of the attachment protein without ablating the fusogenic capability of the envelope.^5^ The attachment protein is modified to prevent binding to the native receptor (blinded) and retargeted by fusing a targeting moiety (for example an antibody) to the ectodomain, such that the targeting moiety is displayed on the surface of the vector particle.^6^^,^^7^ Pseudotyping with modified Nipah viral (NiV) envelope G and F proteins was demonstrated to generate vectors with improved titer compared to the measles envelope; however, the titers are generally much lower than those of VSV-G pseudotyped vectors, resulting in a significant hurdle to the use of these vectors clinically.

Replacing the single-chain variable fragment (scFv)-targeting moiety with a designed ankyrin repeat protein (DARPin) was reported to improve the transduction efficiency of LV vectors pseudotyped with retargeted NiV envelope proteins in the context of CD8^+^ T cell targeting.^7^ Here, we have confirmed and extended this observation using DARPins to CD8 and CD3, and we have shown that variable domain of heavy chain-only antibody (VHH) binders for CD8 can produce LV vectors with a similar efficiency to those generated with the CD8 DARPin. Importantly, we also discovered that we can further enhance the functional titer of these vectors by pseudotyping with a mixture of retargeted and non-targeted, blinded G proteins in addition to the F protein (termed “mixed” envelope).

Using this mixed envelope composition, we generated fourth-generation LV vectors enabling targeted delivery of a CD19-41BB-CD3ζ CAR transgene to T cells using a CD3-specific DARPin or to CD8^+^ T cells using either DARPin or VHH binders for CD8. We assessed the ability of these vectors to directly engineer CAR T cells *in vivo* when injected systemically into the bloodstream of a CD34^+^ cord blood (CB)-engrafted humanized mouse model and compared them to VSV-G pseudotyped vectors in this setting. All the vectors generated very high levels of CAR T cells and mediated rapid and sustained B cell aplasia, albeit with some differences in peak CAR T cell levels and the kinetics of B cell ablation. The retargeted vectors mediated specific T cell transduction with minimal transduction detected in other immune cell types or the liver, in contrast to the VSV-G pseudotyped vector, which additionally transduced murine CD45^+^ (mCD45^+^) cells in the spleen and resulted in high levels of vector copies detectable in the liver.

## Results

### Generation of retargeted LV vectors with improved transduction efficiency

**Figure 1:**
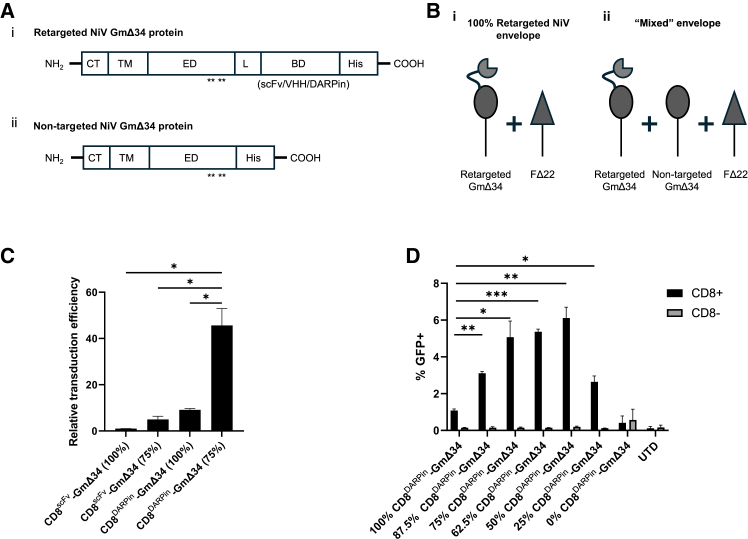
Use of a DARPin and the mixed envelope composition improved the transduction efficiency of retargeted LV vectors pseudotyped with a modified NiV envelope (A) Schematic representation depicting the overall domain organization of the modified Nipah G proteins used for retargeting the LV vectors in combination with a modified Nipah FΔ22 protein (F, not shown). The mixed envelope is composed of a mixture of both (i) the retargeted NiV GmΔ34 protein including a targeting moiety to a cell-specific receptor fused to the carboxy terminus and (ii) the non-targeted NiV GmΔ34 without a targeting moiety. Both versions contain four point mutations to prevent binding to the native targets (E501A, W504A, Q530A, and E533A, indicated with asterisks) and a truncation of the cytoplasmic tail to enhance incorporation into the vector particle, as described previously by Bender et al. CT, cytoplasmic tail; TM, transmembrane domain; ED, ectodomain; L, linker; BD, binding domain; His, His-tag. (B) Schematic representation of the composition of the retargeted NiV envelope (i) and the mixed envelope (ii) composed of a mixture of retargeted NiV GmΔ34 (fused to a targeting moiety) and non-targeted NiV GmΔ34. (C) Impact of the mixed envelope composition on the transduction efficiency of GFP vectors pseudotyped with a retargeted NiV envelope composed of a retargeted CD8-NiV GmΔ34 attachment protein displaying either scFv or DARPin binders for CD8 (CD8^scFv^-NiV and CD8^DARPin^-NiV GmΔ34, respectively). The composition of the G protein component was either 100% retargeted CD8-NiV GmΔ34 or a mixed envelope with 75% retargeted CD8-NiV GmΔ34 and 25% non-targeted NiV GmΔ34 proteins. Transduction efficiency was evaluated by flow cytometry on CD8^+^ T cells 12 days after transduction (*n* = 2). Relative efficiency is shown compared to the CD8^scFv^-NiV 100% vector. (D) Impact of different proportions of retargeted CD8^DARPin^-NiV GmΔ34 and non-targeted NiV GmΔ34 on the transduction efficiency of a GFP LV vector. The proportion of retargeted CD8^DARPin^-NiV GmΔ34 and non-targeted NiV GmΔ34 proteins was varied from 0% to 100% and 100% to 0%, respectively. Flow cytometric analysis of GFP expression in CD8^+^ and CD8^−^ negative populations 12 days after transduction (*n* = 2). Axis labels refer to the proportion of G protein composed of the retargeted CD8^DARPin^-NiV GmΔ34 with the remaining fraction made up of non-targeted NiV GmΔ34. Data shown as mean ± SD. Statistical significance calculated by unpaired two-tailed t test and shown as ∗*p* < 0.05, ∗∗*p* < 0.01, ∗∗∗*p* < 0.001, and ∗∗∗∗*p* < 0.0001. See also Figure S1.

The Nipah paramyxoviral envelope proteins were retargeted as described previously by Buchholz and colleagues.^6^ Briefly, the G protein was mutated at four sites (E501A, W504A, Q530A, and E533A) to prevent binding to its native receptor EphrinB2/B3, a targeting moiety was fused to the carboxy terminus to allow binding to specific target cell types, and the cytoplasmic tail of both G and F proteins were truncated to allow pseudotyping of LV vector particles, herein referred to as GmΔ34 and FΔ22, respectively (Figures 1A and 1B).^6^

DARPins have been shown to mediate improved transduction of CD8^+^ T cells compared to scFv targeting moieties when incorporated into these retargeted LV vectors,^7^ and our experiments also confirmed this observation (Figure 1C). However, the titer of these retargeted vectors was relatively low compared to those pseudotyped with VSV-G. The fusion of the NiV envelope with target cells is thought to be orchestrated by a conformational change upon binding of the attachment G protein to its target receptor, which triggers the F protein to promote fusion.^8^ We hypothesized that, in the case of the retargeted NiV envelopes, the interaction with the F protein might be occluded by the presence of the targeting moiety on the C′ terminus of the attachment protein. We therefore investigated whether the transduction efficiency may be improved by incorporating a portion of non-targeted GmΔ34 proteins into the envelope to create a mixed envelope consisting of both retargeted CD8-NiV GmΔ34 and non-targeted NiV GmΔ34, in addition to the FΔ22 fusion protein (schematic representation in Figure 1B). The overall amount of plasmid transfected encoding the G protein remained the same (0.02 μg per mL) but consisted of 75% retargeted CD8-NiV GmΔ34 (0.015 μg per mL) and 25% non-targeted NiV GmΔ34 (0.005 μg per mL).

We discovered that LV vectors pseudotyped with the mixed envelope utilizing either scFv or DARPin binders for CD8 (CD8^scFv^-NiV and CD8^DARPin^-NiV GmΔ34, respectively^6^^,^^7^^,^^9^) resulted in improved gene transfer to CD8^+^ T cells with an increase in the proportion of cells expressing GFP compared to the equivalent envelope containing 100% retargeted CD8-NiV GmΔ34 (Figure 1C). GFP expression was largely restricted to CD8^+^ T cells for all retargeted vectors, including those with the mixed envelope (Figure S1). We further evaluated different proportions of retargeted CD8^DARPin^-NiV GmΔ34 to non-targeted NiV GmΔ34 from 0% to 100% and identified an optimal range of 50%–75% retargeted CD8^DARPin^-NiV GmΔ34 and 25%–50% non-targeted NiV GmΔ34 (Figure 1D). Specificity was maintained as demonstrated by minimal transduction of cells not expressing CD8 (CD8^−^ population) in all conditions.

### Specific and efficient transduction of CD4^+^, CD8^+^, and CD3^+^ T cells with retargeted LV vectors composed of a mixed NiV envelope with different types of targeting moieties

**Figure 2:**
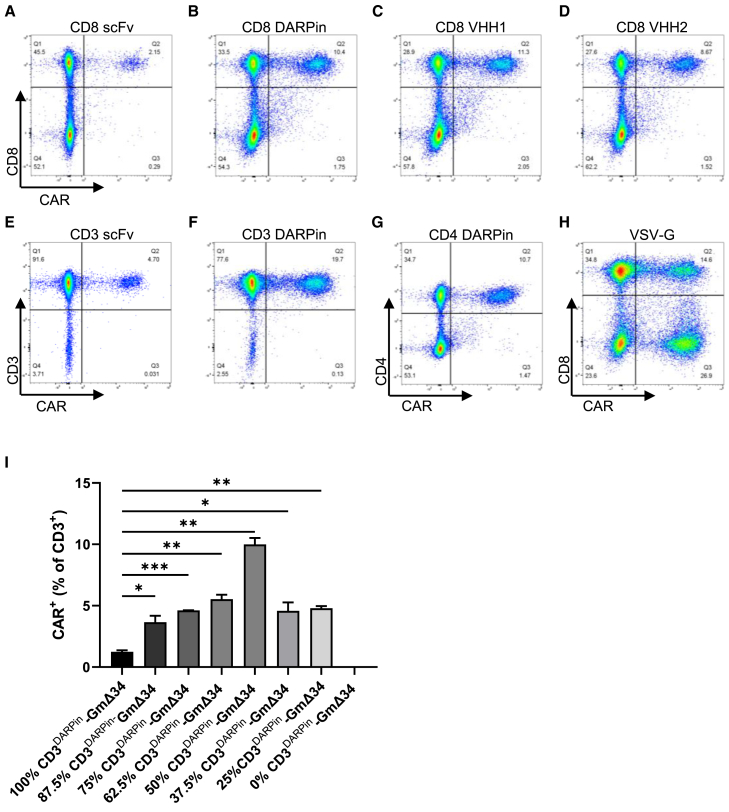
Specific transduction of CD4^+^, CD8^+^, and CD3^+^ T cells with retargeted LV vectors composed of a mixed NiV envelope using scFv, DARPin, or VHH targeting moieties Flow cytometry analysis of T cells transduced with CD19 CAR LV vectors using a mixed envelope with a ratio of 75% retargeted NiV GmΔ34 and 25% non-targeted NiV GmΔ34 incorporating a variety of different targeting moieties. Flow cytometry plots of CAR expression and target receptor expression for T cells transduced with vectors pseudotyped with the following envelopes: (A) CD8^scFv^-NiV, (B) CD8^DARPin^-NiV, (C) CD8^VHH1^-NiV, (D) CD8^VHH2^-NiV (humanized VHH), (E) CD3^scFv^-NiV LV, (F) CD3^DARPin^-NiV LV, (G) CD4^DARPin^-NiV, and (H) VSV-G. Flow cytometry analysis performed 12 days after transduction with matched particle numbers (1.5E+10 RNA copies). (I) Impact of different proportions of retargeted CD3^DARPin^-NiV GmΔ34 and non-targeted NiV GmΔ34 proteins on the transduction efficiency of a CD19 CAR LV vector with a mixed envelope. Axis labels refer to the proportion of retargeted CD3^DARPin^-NiV GmΔ34 as percentage of the overall amount of G protein, with the remaining fraction made up of non-targeted NiV GmΔ34. Flow cytometric analysis of CAR expression in the CD3^+^ population 12 days after transduction (*n* = 2). Data shown as mean ± SD. Statistical significance calculated by unpaired two-tailed t test and shown as ∗*p* < 0.05, ∗∗*p* < 0.01, ∗∗∗*p* < 0.001, and ∗∗∗∗*p* < 0.0001.

The type of targeting moiety used with the NiV retargeting system can influence the functionality of the vectors. DARPins with specificity for CD8 were previously shown to generate vectors with improved titers compared to an scFv.^7^ We speculated that this may be due to their smaller size and reduced propensity for aggregation compared to scFv antibody fragments, and so we hypothesized that the use of single-domain VHH antibody binders might be similarly beneficial for use with the NiV retargeting system. We therefore evaluated two VHH binders for CD8, a camelid VHH (VHH1) and a humanized version (VHH2) (2C8 and 2C8.v144)^10^ for their ability to mediate retargeting of an LV vector encoding an anti-CD19 CAR to enable specific transduction of CD8^+^ T cells. A mixed envelope composed of 75% retargeted CD8-NiV GmΔ34 and 25% non-targeted NiV GmΔ34 (75/25 ratio) was used. The VHH antibodies both generated CD8-specific LV vectors with improved efficiency compared to the scFv and with similar efficiency to vectors retargeted using the CD8 DARPin (Figures 2A–2D).

In order to target all T cell subsets or to specifically target CD4^+^ T cells, we also evaluated DARPin binders for CD3 and CD4 (CD3 DARPin4 and CD4 DARPin 57.2),^11^^,^^12^ which had not been previously tested with the Nipah retargeting system, using the 75/25 mixed envelope described above. In addition, we compared the CD3 DARPin to an scFv (TR66.opt) that was previously demonstrated to mediate T cell transduction with lentiviral vectors pseudotyped using the retargeted Nipah envelope.^13^ We observed that both the CD3 and CD4 DARPins could mediate efficient CAR gene transfer to their respective T cell types (Figures 2E–2G). Furthermore, the CD3 DARPin mediated higher transduction than the CD3 scFv, supporting the utility of DARPins in this setting. All retargeted vectors showed specificity to their target cell types, in contrast to the VSV-G pseudotyped control vector, which, as expected, did not show specificity to a particular population (Figure 2H).

To further assess the impact of the mixed envelope, we generated vectors pseudotyped with different proportions of CD3^DARPin^-NiV GmΔ34 and non-targeted NiV GmΔ34. As observed for vectors pseudotyped with CD8^DARPin^-NiV GmΔ34, the mixed envelope composition improved the efficiency of transduction of target-expressing (CD3^+^) T cells by vectors pseudotyped with different proportions of CD3^DARPin^-NiV GmΔ34 (25%–87.5%) and non-targeted NiV GmΔ34 compared with the vectors comprising 100% CD3^DARPin^-NiV GmΔ34 (Figure 2I). We chose to use a mixed envelope with 75% retargeted NiV GmΔ34 and 25% non-targeted NiV GmΔ34 for all future experiments as this appeared to be in the optimal range for both CD8 and CD3 binders.

### Development of fourth-generation retargeted LV vectors with improved safety profile and minimal CAR incorporation

By utilizing a retargeted envelope to pseudotype the vector, we were able to promote specific targeting of T cells, but it has been shown previously that CD19 CAR protein incorporated into the vector particle can mediate enhanced transduction of malignant B cells^14^ and that inadvertent transduction of leukemia cells can cause masking of the CD19 antigen, resulting in resistance to killing by CD19 CAR T cells.^15^ We therefore evaluated whether a system capable of suppressing the translation of the CAR transgene during vector production could be used to limit the incorporation of CAR proteins into the vector particles pseudotyped with the retargeted, mixed NiV envelope. We used an updated version of the TRiP system, which mediates repression of transgene translation during LV vector production.^16^^,^^17^ This system performs optimally within a fourth-generation LV vector backbone from the TetraVecta system called SupA2KO-LV, which also has several modifications that improve the quality and safety profile of the vector product, providing benefits for direct *in vivo* administration. First, SupA2KO-LV vectors contain a completely synthetic stem loop 2 within the packaging signal, thus removing the major splice donor (MSD) and adjacent cryptic splice donor, which eliminates aberrantly spliced vector RNA species (that express transgene) and promotes the generation of full-length RNA genomes. Production of SupA2KO-LV vectors is dependent on co-expression of a modified U1 small nuclear RNA (snRNA) that binds to the packaging region of the genomic RNA (J.W. et al., unpublished data).^18^^,^^19^ Secondly, the SupA-LTR consists of modified self-inactivating (SIN)-LTRs harboring sequences to improve the efficiency of polyadenylation and transcriptional insulation of the integrated transgene (J.W. et al., unpublished data).^20^

We generated LV vectors encoding a CD19-41BB-CD3ζ CAR within three different genomes: the fourth-generation TetraVecta system SupA2KO-LV vector genome harboring the tryptophan RNA-binding attenuation protein (TRAP)-binding sequence (*tbs*) of the TRiP system within the CAR transgene 5′ UTR (overlapping the Kozak/ATG sequence), and two standard third-generation LV genomes (with an intact MSD), with or without the *tbs*. The LV vectors were pseudotyped with the CD8^DARPin^-NiV mixed envelope and produced in either a TRAP-expressing cell line (NTRP10) or a standard HEK-293T suspension cell line (1.65S) without TRAP. Both the TRAP protein and *tbs* are required for the TRiP system to function.^16^

**Figure 3:**
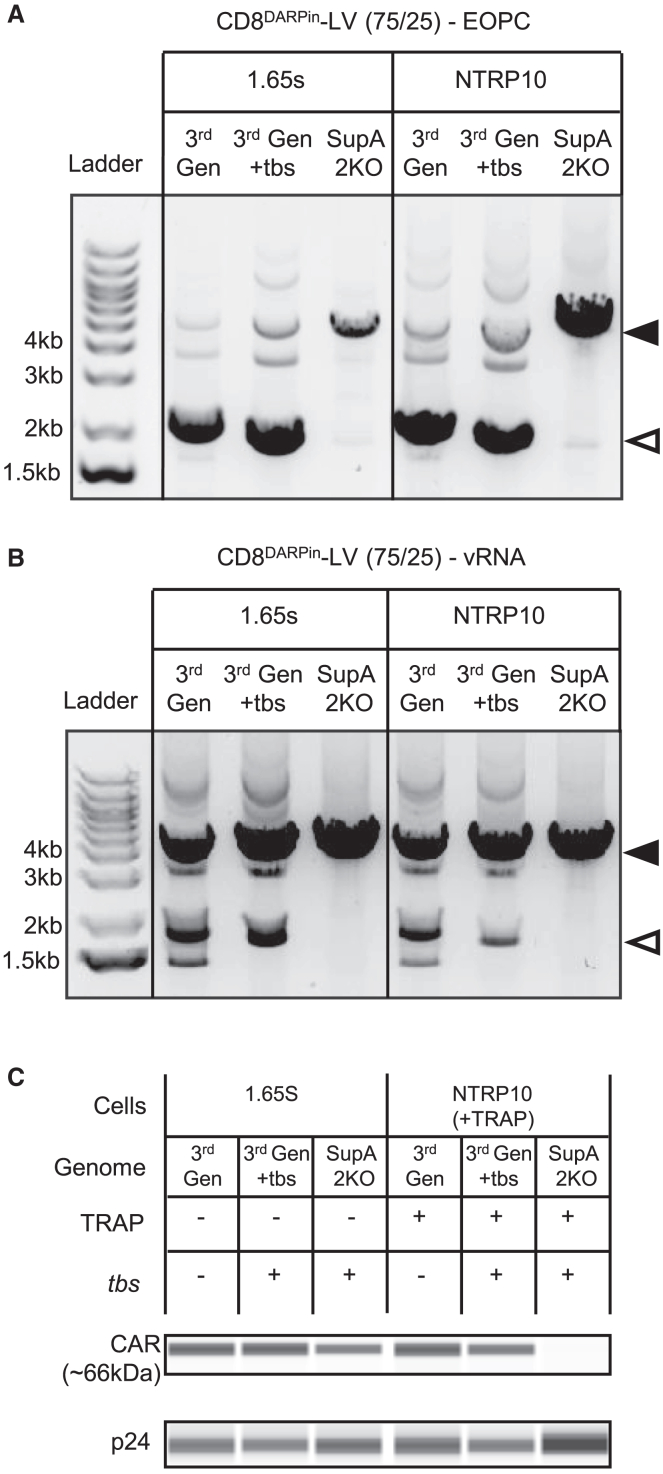
Production of retargeted lentiviral vectors using the SupA2KO-LV genome and TRiP system reduced vRNA splicing and prevented CAR incorporation into the vector particles Vectors were generated using the retargeted NiV mixed envelope with 75% retargeted CD8^DARPin^-LV NiV GmΔ34 and 25% non-targeted NiV GmΔ34 (CD8^DARPin^-LV (75/25)) and the following CD19-41BB-CD3ζ CAR-expressing LV genomes: third-generation HIV sin-LTR genome without *tbs* (3^rd^ Gen), or with *tbs* (3^rd^ Gen + *tbs*), or using fourth-generation TetraVecta system with 2KO genome, SupA-LTR, and *tbs* (SupA 2KO). Vectors were produced in either 1.65S cells (without TRAP) or in NTRP10 cells (with TRAP). (A and B) RT-PCR analysis of splicing of viral genomic RNA (vRNA) transcripts in (A) end of production cells (EOPC) and (B) vector particles. Black arrowhead indicates full-length vRNA; white arrowhead indicates aberrantly spliced vRNA. (C) Western blot analysis of CAR and p24 incorporation into vector particles assessed using the Jess system.

To assess vector genomic RNA integrity, we performed RT-PCR on RNA harvested from cells at the end of production (EOPCs) and vector particles. In other studies, we have shown that the spliced forms of viral genomic RNA (vRNA) transcripts detected in third-generation LV product are efficiently converted to episomal cDNA in target cells (J.W. et al., unpublished data). The traditional paradigm is that only RNA encoding the full packaging signal is expected to be actively packaged into the vector particles. Our data show that, while the proportion of spliced vRNA to full-length vRNA is lower in LV product compared to EOPCs (due to active packaging of full-length vRNA), the spliced RNA is present at readily detectable levels despite only harboring a partial packaging signal (5′ R to MSD). As expected, this semi-quantitative analysis demonstrated virtually complete elimination of aberrantly spliced vRNA transcripts in the EOPC samples when the SupA2KO-LV genome was employed (Figure 3A), resulting in the packaging of solely full-length transcripts in the vector particles in comparison to the third-generation LV genome (±*tbs*) (Figure 3B).

We then assessed whether the TRiP system was able to prevent CAR incorporation into the vector particles. Using an antibody to the CD3ζ domain of the CAR protein, we were able to detect the presence of the CAR protein within vector particles derived from 1.65S cells (cells without TRAP) and from TRAP-expressing NTRP10 cells in the absence of the *tbs*. Interestingly, the CAR was also still detectable in the vectors produced using the third-generation LV genome with the *tbs* even when produced in the presence of TRAP, whereas CAR protein was undetectable in particles produced using the SupA2KO-LV genome with the *tbs* in the presence of the TRAP protein in NTRP10 cells (Figure 3C). The lack of TRiP system activity in the third-generation genome in this case is likely due to potent splicing from the MSD generating excessive levels of CAR-expressing spliced vRNAs. This can vary according to the internal transgene cassette sequences but is typically aggravated by the presence of strong splice acceptors (data not shown). However, the 2KO modification inactivates splicing from the MSD, and therefore these additional CAR-encoding RNAs are absent during LV production, such that the CAR is not detected in the LV vector product. In summary, optimal removal of the CAR protein from the vector particles required both the 2KO modification and TRiP system to be used in combination.

### Efficient generation and expansion of CAR T cells *in vivo* using CD8- and CD3-targeted fourth-generation LV vectors with a mixed NiV envelope

Having successfully identified improvements to both the efficiency and quality of these retargeted vectors *in vitro*, we then wished to assess their ability to generate functional CAR^+^ T cells directly *in vivo*.

We decided to evaluate vectors with the TetraVecta system SupA2KO-LV genome targeted to either CD3 to enable targeting of all T cells, or CD8 to target CD8^+^ T cells, and to compare them to the pantropic VSV-G pseudotyped SupA2KO-LV vector as a control (referred to as VSV-LV). We selected the best-performing binders for evaluation *in vivo*—the DARPin and the humanized VHH for CD8, and the DARPin for CD3—and utilized the mixed NiV envelope composition described above (vectors referred to as CD8^DARPin^-LV, CD8^VHH^-LV, and CD3^DARPin^-LV, respectively).

To generate the larger volume of vector required for the *in vivo* study, we scaled up the vector production using Applikon EZ stirred-tank bioreactors to generate 5 L of crude vector. The crude vector was then purified by anion-exchange chromatography and additionally concentrated ∼1,000-fold by centrifugation to generate the final vector product. The SupA2KO-LVs vectors encoded a CD19-41BB-CD3ζ CAR (using an FMC63 scFv similar to those used commercially) driven by the EF1α promoter with *tbs* overlapping the Kozak/ATG, and they were produced in NTRP10 cells to enable use of the TRiP system. The vectors were pseudotyped with either the mixed NiV envelope targeting CD8^+^ or CD3^+^ T cells (CD8^DARPin^-LV, CD8^VHH^-LV, and CD3^DARPin^-LV) or the standard VSV-G envelope (VSV-LV).

The vectors were titrated on SupT1 cells and a qPCR integration assay was used to define the number of transducing units (TU) per mL (with the exception of the CD3^DARPin^-LV, vector which is unable to transduce this cell line), and RNA copy number analysis was performed to quantify particle numbers (Table S1). To further confirm the functionality of the vectors, we evaluated the transduction efficiency in the target population using peripheral blood mononuclear cells (PBMCs) (Table S1; Figure S2A). The titers were relatively similar for the retargeted vectors, albeit with some differences in the PBMC titer, but, as expected, they were lower than for the VSV-G pseudotyped vector. All the vectors generated CAR T cells with similar cytotoxic activity *in vitro* as assessed by co-culture with CD19^+^ cell lines (Figures S2B–S2D).

**Figure 4:**
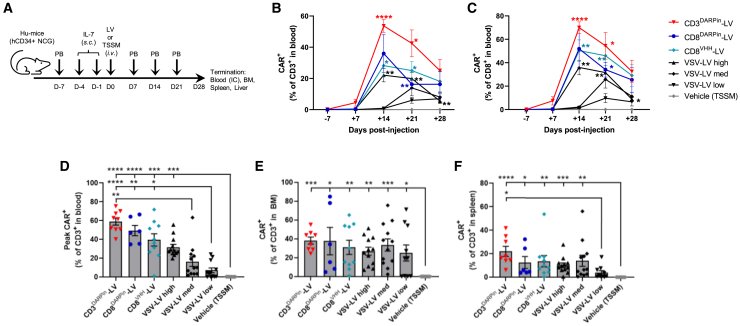
Rapid and efficient generation of CAR T cells *in vivo* by intravenous injection of fourth-generation SupA2KO-LV vectors targeted to CD3^+^ or CD8^+^ T cells (A) Outline of the *in vivo* gene-transfer study. Humanized mice (NCG mice engrafted with human CD34^+^ hematopoietic stem cells) were injected subcutaneously (s.c.) with IL-7 on day (D) −4 and D−1 prior to intravenous (i.v.) injection of 200 μL of LV vectors or TSSM vehicle on D0. Peripheral blood (PB) samples were taken at D−7 to assess humanization and at weekly time points (D7, D14, D21, and D28) after treatment for flow cytometry analysis. Study was terminated at D28 and bone marrow (BM), spleen, and liver were taken for analysis. (B and C) Flow cytometric analysis of proportion of CAR^+^ T cells over time. (B) CAR^+^ % of CD3^+^ cells; (C) CAR^+^ % of CD8^+^ cells. Statistical significance compared to TSSM vehicle control assessed by mixed-effect model with Geisser-Greenhouse correction and Tukey’s multiple comparison test. (D) Peak levels of CAR^+^ T cells generated by each vector (average of the maximum CAR^+^ percentage of CD3^+^ cells for each mouse). (E and F) Proportion of CAR^+^ T cells (% of CD3^+^ cells) in BM (E) and spleen (F) at termination (D28). Data shown as mean ± SEM (*n* = 6–12 per group). Individual data points also shown for (D)–(F). Significant results indicated (Kruskal Wallis test with Dunn’s multiple comparisons). ∗*p* < 0.05, ∗∗*p* < 0.01, ∗∗∗*p* < 0.001, and ∗∗∗∗*p* < 0.0001. See also Figure S2.

Each of the vectors, or vehicle only control (TSSM buffer), were administered by intravenous injection into immunodeficient NOD-*Prkdc*^*em26Cd52*^ *Il2rg*^*em26Cd22*^/Nju (NCG) mice humanized with CD34^+^ cord blood (CB)-derived stem cells (study design summarized in Figure 4A). We chose this model as high levels of both B and T cells are stably generated allowing the monitoring of both CAR T cell generation and activity by flow cytometric analysis of CAR expression and resulting B cell depletion. It also enabled us to assess the specificity of the vectors by analyzing CAR expression in other human immune populations. Compared to mice engrafted with PBMCs, the incidence of potentially confounding xenogeneic graft-versus-host disease is much reduced in this model.^21^ To ensure high T and B cell reconstitution, the mice were treated 20–25 weeks post humanization, and only animals with >40% humanization rate were used for the experiment. A baseline blood sample was taken 1 week prior to injection of the vector. Interleukin (IL)-7 was injected subcutaneously on day (D) −4 and D−1 prior to injection of the vectors (or vehicle), as this cytokine has been reported to enhance the transduction of T cells.^13^^,^^22^^,^^23^ Peripheral blood samples were taken weekly after injection for flow cytometry analysis of immune cells and CAR expression. Flow cytometry was also performed at termination on blood, spleen, and bone marrow (BM) samples, and liver samples were processed for vector copy-number analysis.

The dose in terms of number of TU administered for the CD8^DARPin^-LV and CD8^VHH^-LV vectors was the same (1.8E+07 TU),(Table S1). The titer of the CD3^DARPin^-LV vector could not be assessed using the same cell line as the other vectors; however, the number of particles injected (2.0E+11) was similar to that for the CD8^DARPin^-LV and CD8^VHH^-LV vectors (1.6E+11 and 1.4E+11, respectively), although the functional titer on PBMCs appeared to be slightly lower for this vector (Table S1). We evaluated three doses of the VSV-G vector: undiluted vector was used for the high-dose group, while the medium- and low-dose groups received vector that was diluted 1:8 and 1:16, respectively. The lowest dose of VSV-G pseudotyped vector was selected to match the number of TU administered for the retargeted CD8-LV vectors (1.8E+07 TU; Table S1). However, the physical titers of the retargeted vectors were higher than for the VSV-G pseudotyped vector, and so the number of particles injected for the retargeted vectors was higher than for the VSV-G vector.

Weekly flow cytometric monitoring of CAR expression in the blood revealed that high levels of CAR^+^ CD3^+^ and CD8^+^ T cells were detectable within 14–21 days of injection in all groups injected with retargeted vectors or the high dose of VSV-LV and were significantly above background levels (Figures 4B–4D). The CD3^DARPin^-LV generated the highest levels of CAR^+^ T cells (∼54% of CD3^+^) with an average of 114 cells/μL of CAR^+^ CD3^+^ cells and 67 cells/μL of CAR^+^ CD8^+^ cells in the blood at D14 (Figures S2F and S2G). The two different CD8-targeted vectors (CD8^DARPin^-LV and CD8^VHH^-LV vectors) generated relatively similar levels of CAR^+^ T cells to each other, although slightly less than the CD3^DARPin^-LV vector (Figures 4B–4D, S2F, and S2G). Only the CD3^DARPin^-LV vector generated appreciable numbers of both CD4^+^ and CD8^+^ CAR^+^ cells (Figures S2G and S2H). Despite the higher functional titer of the VSV-G vector, the VSV-LV groups yielded lower numbers of CAR^+^ T cells than the CD3- and CD8-targeted vectors, and this was dose dependent with VSV-LV medium and low-dose groups having significantly lower levels of CAR^+^ T cells than the high-dose group at D7–D14 (Figure 4B, D7 *p* < 0.05, D14 *p* < 0.01), presumably due to different specificities of the vectors leading to uptake of the VSV-G vector by other cell types *in vivo*.

The frequency of CAR T cells was very low at D7, but, by D14–D21, a very high proportion of T cells were CAR^+^, suggesting that, after transduction, the CAR T cells expand considerably *in vivo* for 2–3 weeks before contracting. The highest average frequency of CAR T cells was observed at D14 for all vector-treated groups except the low- and medium-dose VSV-LV groups, which were slower to reach maximal expansion, with the medium-dose group average peaking at D21 and the low-dose group continuing to increase to D28 when the animals were terminated (Figures 4B and 4C). The peak level of CAR T cells was not significantly different between the high and medium doses of VSV-LV despite 8-fold less vector having been administered (Figure 4D), suggesting that lower doses of vector could generate similar levels of CAR T cells but with delayed kinetics. The maximal expansion of CAR T cells may therefore be limited by other factors than the initial transduction rate, such as the amount of target antigen or cytokine levels.

CAR T cells were also detected at high frequency in the BM and spleen at termination on D28, with relatively similar levels of CAR^+^ CD3^+^ cells generated by the different vectors (Figures 4E and 4F). The impact of the different doses of VSV-LV was less apparent in the BM and spleen, presumably due to the longer expansion phase in the low- and medium-dose groups and the regression of CAR^+^ T cells in the high-dose group by this time, as was observed in the blood analysis at D28 (Figures 4B and 4C). The proportion of CAR^+^ T cells in the BM was notably higher in all vector-treated groups than in the spleen and blood at D28, perhaps due to ongoing lymphopoiesis leading to continual regeneration of CD19^+^ B cells from stem/progenitors in the BM, thus potentially driving sustained CAR T cell stimulation and expansion in this tissue even after B cell ablation had occurred in the periphery and secondary lymphoid organs.

### Effective killing of B cells and induction of B cell aplasia by CAR T cells generated *in vivo* using fourth-generation LV vectors with a mixed NiV envelope

**Figure 5:**
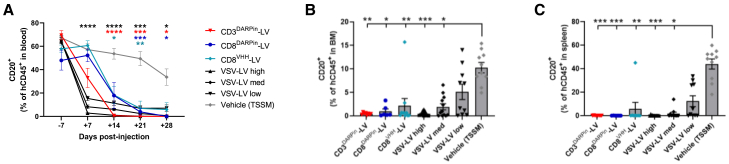
CAR T cells generated *in vivo* using fourth-generation retargeted SupA2KO-LV vectors induce rapid and sustained B cell aplasia (A) B cell levels in the blood over time. For simplicity, only significant results in comparison to vehicle/TSSM are indicated, colors correspond to the groups as shown in the legend (mixed-effects analysis). (B and C) B cell levels in BM (B) and spleen (C) analyzed after termination (D28). Significant results shown for comparison to vehicle/TSSM group (Kruskal-Wallis with Dunn’s multiple comparisons test). Data shown as percentage of CD20^+^ B cells in the hCD45^+^ cell population; mean ± SEM (*n* = 6–12 per group). Individual data points also shown for (B) and (C). ∗*p* < 0.05, ∗∗*p* < 0.01, ∗∗∗*p* < 0.001, and ∗∗∗∗*p* < 0.0001. See also Figure S3.

We assessed the depletion of B cells as a measure of CAR T cell activity since the CD19 CAR was expected to mediate killing of human B cells in this model. We used CD20 as a marker of B cells to avoid any potential issues with under-detection of B cells due to masking or downregulation of the CD19 target antigen. As B cell engraftment tends to drop slightly over time in this humanized mouse model, it was important to compare the depletion at each time point to the vehicle control. The proportion of B cells dropped significantly compared to vehicle in all vector-treated groups within 14–21 days after injection, such that severe B cell aplasia was apparent by D28 in all vector-treated groups treated with the retargeted vectors and in the high-dose VSV-LV group (Figure 5A) (<1% of CD20^+^ B cells remaining in most animals, except a single outlier in the CD8^VHH^-LV group that also had low/undetectable CAR T cells [<1%]; Figure 4D).

The kinetics of B cell aplasia were slightly different between the different vectors, with the VSV-LV groups having a more rapid onset of depletion than the other groups with a significant drop in B cells apparent by D7, even before CAR T cells were readily detectable (Figure 5A, *p* < 0.0001 for all VSV-LV groups compared to the vehicle control). None of the retargeted vectors had a significant impact on B cell levels at this time point. Within the retargeted vector-treated groups, the CD3^DARPin^-LV vector impacted B cell levels slightly earlier than the CD8-targeted LV vector groups, with levels dropping to <1% by D21 in all animals in the CD3^DARPin^-LV group and by D28 in the CD8^DARPin^-LV and CD8^VHH^-LV groups (except for one outlier in the CD8^VHH^-LV group). For the VSV-LV groups, the kinetics of B cell depletion were slower for the low- and medium-dose groups, with significantly less severe B cell depletion than the VSV-LV high-dose group at D14 (*p* < 0.01), although there was no statistical difference later, thus mirroring the observations for the peak CAR T level.

Significant B cell aplasia was also observed in the BM and spleen at termination (D28) in all vector-treated groups, except the VSV-LV low-dose group, where some animals had higher levels of B cells remaining (Figures 5B, 5C; S3A–S3C). The absolute number of B cells detected in the blood and spleen was several orders of magnitude lower than the vehicle control, such that they were barely detectable. Although B cells were significantly depleted in the BM, slightly higher numbers of B cells remained detectable in this tissue than in the blood and spleen, presumably due to ongoing lymphopoiesis from engrafted hematopoietic stem/progenitor cells within the BM niche.

In summary, all the vectors rapidly generated functional anti-CD19 CAR T cells *in vivo* that expanded and successfully ablated B cells in the circulation and in primary and secondary lymphoid organs within 2–3 weeks of injection.

### Specific T cell targeting *in vivo* with CD8- and CD3-targeted envelopes compared to VSV-G

**Figure 6:**
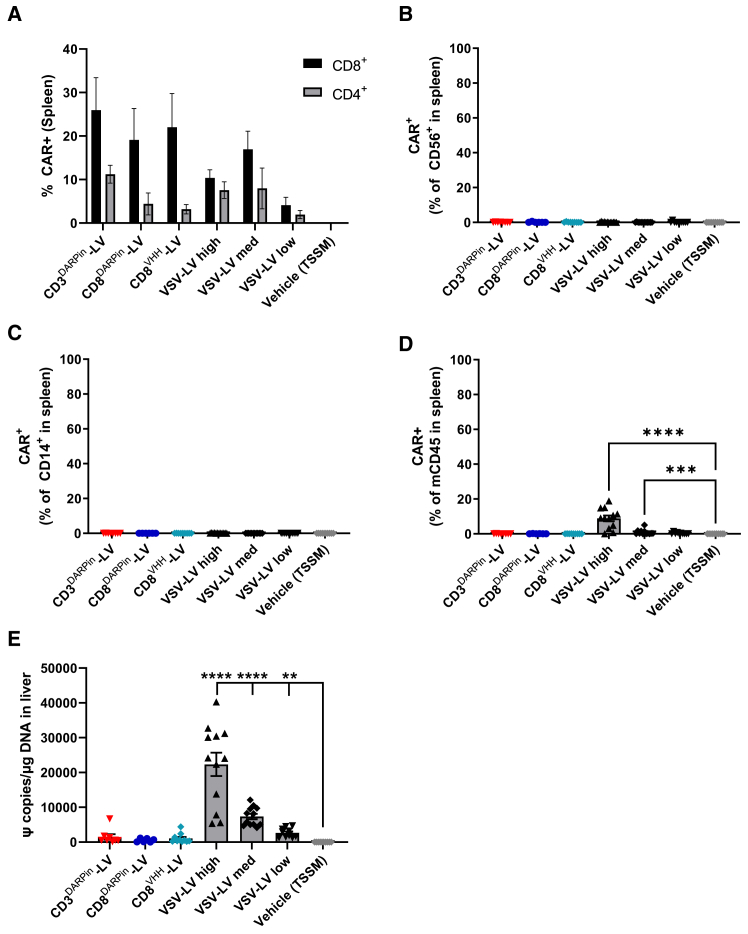
Specific targeting of T cells *in vivo* with fourth-generation retargeted SupA2KO-LV vectors (A) Proportion of CAR^+^ CD8^+^ and CD4^+^ T cells in the spleen. (B–D) Assessment of CAR expression in other immune cells in the spleens of the animals. Proportion of CAR^+^ (B) CD56^+^ NK cells, (C) CD14^+^ monocytes, and (D) mCD45^+^ cells. (E) Vector copies per μg of DNA in the liver assessed by qPCR. Data shown as mean ± SEM (*n* = 6–12 per group); individual data points also shown for (B)–(E). Statistical analysis performed using Kruskal-Wallis with Dunn’s multiple comparison test. ∗*p* < 0.05, ∗∗*p* < 0.01, ∗∗∗*p* < 0.001, and ∗∗∗∗*p* < 0.0001. See also Figure S4.

We evaluated the specificity of the targeted vectors by flow cytometric analysis of CAR expression in immune cell populations in the blood, BM, and spleen (Figures 6A–6D and S4A–S4G). CAR expression mediated by all the retargeted vectors was predominantly restricted to CD3^+^ T cells with minimal expression detected in the other human immune cell populations analyzed (natural killer [NK] cells, monocytes, or B cells). CD8-targeted vectors resulted in CAR expression that was largely restricted to CD8^+^ T cells, although a low frequency of CAR^+^ cells was detected in CD4^+^ T cells in a few animals (Figures 6A; S4A) possibly due to transduction of double-positive CD8^+^CD4^+^ precursor cells. CAR expression resulting from transduction with the non-targeted VSV-G pseudotyped vectors was also largely confined to T cells, with minimal expression detected in other human immune cell types; however, in animals treated with the high dose of VSV-LV, a high frequency of CAR^+^ cells was additionally observed within the mouse CD45^+^ compartment in the spleen, although not in the BM or blood (Figures 6D, S4D, and S4G). These cells are likely to be myeloid cells since no murine lymphoid cells are generated in the NCG mouse model used. We also observed that VSV-G pseudotyped vectors could mediate high levels of transduction of monocyte-derived macrophages *in vitro*, whereas CD8^DARPin^-NiV pseudotyped LV vector failed to transduce these cells even at high MOI (Figure S4H).

The liver is known to act as a “sink” for viral vectors pseudotyped with VSV-G.^24^^,^^25^^,^^26^ We therefore further evaluated the specificity of the retargeted vectors by analyzing integrated vector copy number (VCN) in the liver of treated animals. As expected, we detected high levels of vector within the livers of the mice treated with VSV-G pseudotyped vector; moreover, a dose response was observed, with VCN decreasing as VSV-LV vector dose decreased (Figure 6E). On the contrary, the retargeted vectors showed minimal evidence of liver transduction, with very low levels of vector copies detected.

## Discussion

In this report, we describe a highly efficient retargeted NiV envelope system that overcomes the hurdles of previous engineered envelopes and combined this with a fourth-generation lentiviral vector platform called the TetraVecta system. We have shown that vectors generated with this system are capable of highly efficient and specific T cell transduction *in vivo*, leading to generation and expansion of large numbers of CAR T cells and induction of rapid B cell aplasia. These vector particles have improved efficiency due to the incorporation of a mixed envelope composed of both retargeted and non-targeted NiV GmΔ34 proteins, in addition to the NiV FΔ22 protein, and the use of compact targeting moieties (DARPin or VHH) displayed on the retargeted NiV GmΔ34 protein. In addition, these fourth-generation vectors are anticipated to have an improved safety profile due to modifications to the viral genome that eliminate aberrantly spliced vRNA production and incorporation into LV product, improve transgene transcriptional insulation, and substantially reduce expression of the CAR during vector production.

To ensure specific transduction of target cell types, it is important to prevent the CAR protein from being incorporated into the vector particle as this could mediate off-target transduction of CD19-expressing B cells, including tumor cells.^14^ This is undesirable as it has been demonstrated that the inadvertent transduction of a single leukemia cell was capable of mediating relapse in a patient due to the masking of the CD19 antigen by the CAR.^15^ Furthermore, the presence of CAR protein in the vector or peptides displayed on major histocompatibility complex (MHC) class I molecules on the vector could potentially induce an adaptive immune response to the transgene, which could restrict CAR T cell persistence.^27^ We therefore employed the TRiP system to suppress expression of the CAR transgene during vector production to prevent incorporation of the CAR into the vector particles.^16^ Here, we compared a standard third-generation LV to the SupA2KO-LV genome from the TetraVecta system to demonstrate and confirm other studies (J.W. et al., unpublished data) showing that this fourth-generation LV platform is optimal for maximal utilization of the TRiP System. The degree of aberrant splicing observed from the MSD within third-generation LV genome expression cassettes can vary according to internal transgene sequences, with either obvious splice acceptors (such as that of the EF1α intron) or cryptic acceptors being used, despite the presence of the regulatory protein Rev (J.W. et al., unpublished data). Such spliced vRNAs encode and express the transgene, and therefore an alternative approach of employing a tissue-specific promoter would be unlikely to prevent transgene protein expression during production.

It has been demonstrated that, when using modified Nipah envelopes to retarget lentiviral vectors to CD8^+^ T cells, DARPins tend to produce vectors with greater transduction efficiency than scFv binders.^7^ Here, we confirmed and extended this observation using DARPins to target CD3 as well as CD8, and we further discovered that VHH binders for CD8 generated vectors with similar functional titers to those using the CD8 DARPin. This suggested that VHH binders may be similarly beneficial to DARPins in this setting, perhaps due to their small size compared to scFv and reduced propensity for aggregation. We were able to further enhance the functional titer of these vectors using a mixed envelope composed of a mixture of retargeted and non-targeted blinded Nipah G with an optimal ratio of around 50%–75% retargeted NiV GmΔ34 and 25%–50% non-targeted NiV GmΔ34 (in addition to the NiV FΔ22 protein). We hypothesized that reducing both the size and number of binders displayed on the G proteins may improve the functionality of the vectors by limiting the steric hindrance that could impede the interaction between the G and F proteins that is required to trigger membrane fusion.

Surprisingly, we discovered that, despite the lower functional titer of the retargeted vectors *in vitro*, they could generate higher levels of CAR T cells *in vivo* compared to the VSV-G pseudotyped vector. Furthermore, the CD3-targeted vectors generated a higher level of CAR T cells than the CD8-targeted vectors, including higher levels of CD8^+^ CAR T cells and a slightly more rapid ablation of B cells, suggesting that pan-T cell targeting may be beneficial. However, the differences between the CD3- and CD8-targeted vectors were not significant and may reflect the slightly higher number of particles administered for the CD3^DARPin^-LV vector. Despite differences in the number of CAR T cells generated, the overall B cell aplasia was similar after 3–4 weeks with all vectors, including the lower doses of VSV-G vectors, suggesting that we had exceeded the minimal efficacious dose. Future studies will be required to assess the impact of lower doses of the retargeted vectors on CAR T cell generation and B cell depletion. Furthermore, it will be important to define the minimum dose of vector required for the *in vivo*-generated CAR T cells to ablate tumor cells in an appropriate model prior to moving into clinical studies in patients with lymphoid malignancies. However, these data suggest that the dose of vector administered to patients could be minimized while achieving similar target cell killing, which could reduce the severity of adverse events associated with CAR T cell expansion, such as cytokine release syndrome.

It has been suggested that a combination of CD3 and co-stimulatory receptor engagement may be beneficial for *in vivo* CAR T cell generation leading to the development of vectors displaying both CD3-binders and co-stimulatory molecules.^28^ However, we observed very high levels of CAR T cell generation with vectors pseudotyped with VSV-G or CD8-targeted NiV envelopes, which do not engage the T cell receptor (TCR), suggesting that T cell activation via the TCR is not necessary for efficient T cell transduction *in vivo*. Furthermore, the CD3-targeted vector did not include additional co-stimulatory molecules but was able to generate extremely high levels of CAR T cells. It is worth noting, however, that we pre-conditioned the mice with IL-7 similarly to previous *in vivo* studies evaluating CD3- and CD8-targeted LV vectors.^29^ IL-7 has been shown to improve T cell transduction of resting T cells by overcoming a variety of restriction mechanisms, and so this could have overcome the need for stimulation of the TCR for efficient transduction.^22^^,^^23^^,^^30^^,^^31^ The impact of IL-7 pre-treatment will be assessed in future studies to determine whether it would be beneficial as a pre-conditioning agent for *in vivo* CAR T therapeutics.

It was interesting that, despite the higher functional titer *in vitro*, the VSV-G pseudotyped vector yielded lower levels of CAR T cells *in vivo* than the CD3- or CD8-targeted vectors. This was likely due to uptake of the VSV-G pseudotyped vector by cells other than T cells due to broader expression of the target receptor (low-density lipoprotein receptor, LDLR).^32^ In support of this, we observed high vector copies in the liver and CAR expression in murine CD45^+^ cells in the spleen only with the VSV-G pseudotyped vectors.

Despite the lower levels of CAR T cells generated by the VSV-G pseudotyped vector, the ablation of B cells was notably more rapid than observed with the retargeted vectors, although the overall level of B cell ablation was similar with all vectors after 3–4 weeks. Surprisingly this ablation occurred within 7 days of administration before CAR T cells had expanded, suggesting that an alternative cell type may mediate this early killing. In general, we did not detect appreciable transduction of any human immune cell types other than T cells with any of the vectors (including the VSV-G vector); however, there was significant transduction of murine CD45^+^ cells (presumably myeloid cells) in the spleens of animals treated with the VSV-G vectors, and high vector copy numbers were detected in the livers of animals treated with VSV-G vectors, which was not apparent with the retargeted vectors. Others have previously noted the presence of transduced Kupffer cells and macrophages in the liver and spleen after injection of lentiviral vectors.^28^^,^^33^^,^^34^ Taken together, these observations lead us to speculate that CAR-expressing myeloid cells residing in the spleen and/or the liver may mediate the early killing of B cells seen with the VSV-G pseudotyped vectors. However, it has been previously reported that transduction of antigen-presenting cells (APCs) such as Kupffer cells and splenic macrophages leads to priming of an immune response to the transgene, which is detrimental to the survival of transduced cells.^27^ It may therefore be advisable to avoid potential transduction of myeloid APCs by utilizing retargeted vectors for specific T cell transduction such as those described here.

In summary, we have successfully improved and combined LV-retargeting envelopes with a fourth-generation LV platform for the delivery of CAR transgenes specifically to T cells with high efficiency in animal studies. These improvements will enable the implementation and feasibility of *in vivo* CAR T therapy, which should improve accessibility of these types of curative therapies for patients by removing the need for *ex vivo* manufacturing of CAR T cells.

## Materials and methods

### Cell culture

HEK-293T suspension cell line (1.65S) and a derivative of this cell line (NTRP10) stably expressing TRAP were cultured in Freestyle 293 Expression medium (Gibco, Thermo Fisher Scientific, Altrincham, UK) supplemented with 0.1% Cholesterol Lipid Concentrate (Gibco, Thermo Fisher Scientific, Altrincham, UK). SUP-T1 cells were cultured in RPMI1640 GlutaMAX (Thermo Fisher Scientific, Altrincham, UK) supplemented with 10% heat-inactivated fetal bovine serum (FBS) (TCS Biosciences, Buckingham, UK). Human PBMCs were obtained from NHSBT, with informed consent for use in research and study approved by the East of England Cambridge Central Research Ethics Committee. PBMCs were cultured in X-VIVO 15 medium (Lonza Bioscience, Slough, UK) supplemented with 2.5% human serum (Sigma), 2 mM GlutaMax (Thermo Fisher Scientific, Altrincham, UK), 20 mM HEPES (Thermo Fisher Scientific, Altrincham, UK), 1 mM sodium pyruvate (Merck Life Science, Gillingham, UK), 1% MEM Eagle vitamin mix (Scientific Laboratories Supplies, Nottingham, UK), 10 ng/mL human IL-7 (Miltenyi Biotec, Bisley, UK), and 10 ng/mL human IL-15 (Miltenyi Biotec, Bisley, UK). PBMC activation was performed using Human T-Activator CD3/CD28 Dynabeads (Thermo Fisher Scientific, Altrincham, UK) following the manufacturer’s instructions.

### Vector production

Vector production was performed in 1.65s or NTRP10 suspension cell lines. A total of 1.316 μg DNA per mL of cell suspension was incubated with Freestyle medium and Lipofectamine 2000 CD (Invitrogen, Thermo Fisher Scientific, Altrincham, UK) at 9.5:1:0.6:0.7:1.36 mass ratios of each plasmid (genome:gag/pol:rev:envelope:U1-256). Packaging plasmids and the fourth-generation SupA2KO LV genome and U1-256 plasmids have been described previously.^18^^,^^19^^,^^20^^,^^35^ Envelope plasmids were obtained by replacing the VSV-G sequence with NiV-FΔ22AU1 and NiV-GmΔ34His sequences, as previously described^6^ (GeneArt, Thermo Fisher Scientific, Altrincham, UK) using PspOMI and XhoI restriction enzymes. The different targeting domains have been synthesized and cloned into NiV-GmΔ34His using SgrAI and XhoI restriction enzymes (Genewiz, Azenta Life Sciences, Abingdon, UK). Targeting domains used were as follows: CD8, an scFv based on OKT8,^9^ DARPin 53F6,^7^ and VHH antibodies 2C8 and 2C8.v144^10^; CD3, scFv TR66.opt^13^ and DARPin 4^12^; and CD4, DARPin 57.2.^11^ For the Nipah envelope, plasmids encoding NiV-FΔ22AU1 and retargeted NiV-GmΔ34His were used at 0.05 μg per mL and 0.02 μg per mL, respectively. For the mixed envelope, the total amount of plasmid encoding for the G protein was the same (0.02 μg per mL) but was composed of different proportions of plasmid encoding the retargeted GmΔ34 and non-targeted GmΔ34 proteins. In most cases, unless otherwise specified, the G protein was composed of 75% retargeted GmΔ34 and 25% non-targeted GmΔ34, corresponding to 0.015 μg per mL and 0.005 μg per mL of each plasmid. Vector production was induced 24 h after transfection by the addition of 10 mM (final concentration) sodium butyrate (Merck Life Science, Gillingham, UK) and optionally 250 nM Ingenol-3-angelate (I3A) (Merck Life Science, Gillingham, UK).^36^ Supernatant containing crude vector was collected 24 h after induction and processed differently according to the scale of vector production. Small-scale preparations (up to 30 mL) were clarified and concentrated 100-fold by centrifugation at 6,000 × *g* overnight at 4°C on a 20% sucrose cushion. Large-scale preparations (5 L) used for the *in vivo* study were performed in the Applikon EZ stirred-tank bioreactor, treated with Benzonase (Merck Life Science, Gillingham, UK), and clarified and purified using anion exchange chromatography on an AKTA Pure (Cytiva, Amersham, UK) using the Mustang Q XT5 membrane (Cytiva, Amersham, UK); eluted fractions were treated with Salt Activated Nuclease (ArcticZymes Technologies ASA, Tromso, Norway), diluted to 300 mM NaCl, and concentrated 1,000-fold by centrifugation as detailed above. Vector pellets were finally resuspended in TSSM buffer (sodium chloride [100 mM], Tris, pH 7.3 [20 mM], sucrose [10 mg/mL], and mannitol [10 mg/mL]), aliquoted, and stored at −80°C.

### Vector titration and analysis

Concentrated vectors were used for determining transduction efficiency and specificity *in vitro*. SupT1 cells or activated PBMCs were transduced with serial dilutions of the vector in duplicate and in the presence or absence of nevirapine (Merck Life Science, Gillingham, UK). Flow cytometry was performed on PBMCs on the day of transduction and at least 10 days post transduction; for SupT1, it was performed at least 7 days post transduction using an Attune NxT Flow Cytometer (Thermo Fisher Scientific, Altrincham, UK). Functional titer was assessed either by flow cytometry analysis (biological titer) or qPCR assay (integration titer).

For flow cytometric analysis, staining was performed using CAR FMC63 Idiotype Antibody VioBright515 (Miltenyi Biotec, Bisley, UK) or biotinylated CD19 CAR detection reagent (Miltenyi Biotec, Bisley, UK) with PE Streptavidin (BD Biosciences, Wokingham, UK), anti-CD3 PE (BioLegend, London, UK) or anti-CD3 PerCP-Cy5.5 antibody (BD Biosciences, Wokingham, UK), anti-CD4 BV510 (BioLegend, London, UK) or anti-CD4 AF488 antibody (BioLegend, London, UK), and anti-CD8 PE-Cy7 (BioLegend, London, UK), and finally cells were resuspended in stain buffer with SYTOX AADvanced Dead Cell Stain (Thermo Fisher Scientific, Altrincham, UK). Flow cytometry was performed using an Attune NxT Flow Cytometer (Thermo Fisher Scientific, Altrincham, UK) and analyzed using FlowJo (BD Life Sciences, Wokingham, UK).

To assess integration titer in SupT1 cells (TU per mL), genomic DNA was extracted from the transduced cells 7 days after transduction. DNA extraction was performed on a QIAcube HT instrument using the QIAamp 96 DNA QIAcube HT Kit (QIAGEN, Manchester, UK). Integrated vector genomes were quantified by duplex qPCR using primers and probe sets specific for HIV-1 packaging signal (forward, 5′-TGGGCAAGCAGGGAGCTA-3ʹ; reverse, 5′-TCCTGTCTGAAGGGATGGTTGT-3′; probe, 5′-FAM-AACGATTCGCAGTTAATCCTGGCCTGTT-TAMRA-3′) and a cellular gene, ribonuclease P RNA component H1 (RPPH1) (forward, 5′-CCTAGTCTCAGACCTTCCCAAG-3′; reverse, 5′-GCGGAGGGAAGCTCATCAG-3′; probe, 5′-VIC-CCACGAGCTGAGTGCGTCCTGTCA-TAMRA-3′) on a QuantStudio 6 Flex real-time PCR system (Thermo Fisher Scientific, Altrincham, UK).

To determine the physical titer, RNA was extracted from the lentiviral vectors using the QIAamp Viral RNA Mini Kit (QIAGEN, Manchester, UK) with DNase treatment, reverse transcribed, and vector RNA copy number was quantified by qPCR using the same primers and probe for the HIV-1 packaging signal as above. Vector particle numbers were approximated from RNA copy number assuming two copies of viral RNA per particle.

### Characterization of fourth-generation vectors

Expression of the CD19 CAR transgene in vector particles was evaluated using the Jess western blot system (ProteinSimple/Bio-Techne, Abingdon, UK) and analyzed using the companion Simple Western software. Prior to loading, vectors were diluted according to their RNA copy number to match the number of vector particles in each sample. The primary antibodies used were anti-CD3ζ (ab226475, Abcam, Cambridge, UK) and anti-p24 (ab63913, Abcam, Cambridge, UK). Peak area was used to determine presence of signal, and the blot was used to visualize the data.

To evaluate aberrant splicing of the vector genome, RT-PCR was performed on RNA harvested from EOPCs and vector particles. RNA was extracted from all vectors using the QIAamp Viral RNA Mini Kit (QIAGEN, Manchester, UK) and from EOPCs using the QIAGEN RNeasy Mini Kit (QIAGEN, Manchester, UK) according to manufacturer’s instructions. cDNA was synthesized by treating ∼500 ng of RNA with ezDNase (Thermo Fisher Scientific, Altrincham, UK), followed by addition of DTT for 5 min. Oligo d(T) and dNTPs were added for 5mins at 65°C followed by DTT, SuperScript IV reverse transcriptase (Thermo Fisher Scientific, Altrincham, UK), and a ribonuclease inhibitor (55°C 10 min followed by 80°C for 10 min). Finally, RNase H was added for 20 min at 37°C to complete the reaction. cDNA was diluted 1:20 and amplified with primers designed to span the HIV packaging sequence through to the woodchuck hepatitis virus posttranscriptional regulatory element (WPRE) (JW32, 5′-AAAGCGAAAGGGAAACCAGAG-3′ and JW58, 5′-AGCAGCGTATCCACATAG-3′). PCR products were separated on a 0.75% agarose gel and visualized under a UV light.

### *In vivo* gene-transfer study

The *in vivo* gene-transfer study (protocol number 23Q1038) was carried out at TransCure bioServices (Archamps, France) using female NOD-Prkdc^em26Cd52^ Il2rg^em26Cd22^/Nju (NCG) immunodeficient mice humanized with CD34^+^ hematopoietic stem cells isolated from human cord blood. Only mice with a humanization rate (hCD45/total CD45) above 40% at the engraftment check (at least 20 weeks post humanization) were included in the study. Mice were distributed into groups by stratified randomization, ensuring equal representation of the different CD34^+^ donors in each group. Animals were housed in groups of 2–6 in ventilated cages under standard animal care conditions with water and food available *ad libitum* and monitored daily. All procedures were reviewed and approved by the local ethics committee (CELEAG).

Human IL-7 was prepared in sterile PBS without Mg^2+^/Ca^2+^ and injected into mice via the subcutaneous route at D−1 and D−4 of the study. Lentiviral vectors or vehicle (TSSM) were injected intravenously via tail vein (200 μL) on D0. Body weight and clinical score were monitored three times per week from D−7 to D26. Blood was collected into K3-EDTA tubes by retro-orbital (r.o.) route before the IL-7 injections (D−7) and weekly (D7, D14, and D21) after lentiviral vectors injection, and then by intracardiac puncture (IC) at final takedown (D28). At sacrifice, spleen, liver, and BM were also collected for further analysis.

Vector information for each treatment group is shown in Table S1. We aimed to use the maximum tolerated dose for the retargeted vectors; however, some initial deaths were observed immediately after injection with the highly concentrated retargeted LV vectors (CD3^DARPin^-LV, three animals; CD8^DARPin^-LV, three animals; CD8^VHH^-LV, two animals). We therefore diluted the CD8-targeted vectors for injection into the remaining mice (1:2 dilution), resulting in no further deaths and no further significant impacts on the overall health of the animals in vector-treated groups compared to the vehicle (Figure S2E). One mouse from each of the VSV-LV low-dose and TSSM/vehicle control groups was found dead on D26 and D2, respectively, and one mouse from each of the CD8^DARPin^-LV, CD3^DARPin^-LV, VSV-LV low-dose, and TSSM/vehicle control groups was euthanized for ethical reasons on D14, D17, D8, and D10, respectively. All remaining mice were euthanized at the end of the study on D28. The composition of the groups with the number of mice remaining on D3 and the dose of vectors administered are indicated in Table S1.

### Flow cytometry on blood, BM, and spleen

Blood samples were collected at baseline (D−7), and at 7, 14, 21, and 28 days post vector administration. BM and spleen samples were collected at day 28. Flow cytometry was performed on an Attune NxT Flow Cytometer (Thermo Fisher Scientific, Altrincham, UK) using the following antibodies: anti-CD3 BV421 (BioLegend, London, UK), anti-CD4 APC-R700 (BD Biosciences, Wokingham, UK), anti-CD8a APC (BD Biosciences, Wokingham, UK), anti-CD20 PE-Dazzle594 (BioLegend, London, UK), anti-CAR CD19 biotinylated (Miltenyi Biotec, Bisley, UK), anti-biotin PE (Miltenyi Biotec, Bisley, UK) anti-CD14 Starbright Yellow 800 (Bio-Rad, Watford, UK), anti-HLA-DR APC-Fire810 (BioLegend, London, UK), anti-CD56 PerCP-Vio700 (Miltenyi Biotec, Bisley, UK), anti-CD62L BB515 (BD Biosciences, Wokingham, UK), anti-mCD45 BV786 (BD Biosciences, Wokingham, UK), anti-hCD45 BV650 (BD Biosciences, Wokingham, UK), anti-CD11b BV605 (BioLegend, London, UK), anti-CD11b StarBright Violet 710 (Bio-Rad, Watford, UK), and viability stain FVS510 (BD Biosciences, Wokingham, UK). Samples were analyzed using FlowJo (BD Life Sciences, Wokingham, UK).

### Quantification of VCN by qPCR

Liver was harvested at the end of the study and stored in RNAlater (Thermo Fisher Scientific, Altrincham, UK) for 24 h at 4°C, after which it was removed and tissue was stored at −80°C. Liver sections were lysed using TissueLyser (QIAGEN, Manchester, UK) and DNA was extracted using DNeasy Blood and Tissue Kit (QIAGEN, Manchester, UK).

qPCR was performed using TaqMan Gene Expression Master Mix (Applied BioSystems, Thermo Fisher Scientific, Altrincham, UK) and primers/probe sets to detect HIV ψ packaging signal (forward, 5′-TGGGCAAGCAGGGAGCTA-3ʹ; reverse, 5′- TCCTGTCTGAAGGGATGGTTGT-3′; probe, 5′-FAM-AACGATTCGCAGTTAATCCTGGCCTGTT-TAMRA-3′). Standard curves were prepared using a custom gBlock (Integrated DNA Technologies, Leuven, Belgium) containing the target amplicons. The reactions were run in QuantStudio 6 or QuantStudio 7 Real-Time PCR System (Thermo Fisher Scientific, Altrincham, UK) and analyzed with the associated software.

### Cytotoxicity assay

Activated T cells were transduced with relevant vectors at 1:120 dilution (retargeted vectors) or 1:1,080 dilution (VSV-G vector), cultured for 13 days, and then co-cultured with CD19^+^ target cells lines (RAMOS, NALM6, and MEC1) at effector:target (E:T) ratio of 2 or 0.5 based on total T cell number. After 24 h, the number of live target cells was assessed by flow cytometry (Attune NxT, Thermo Fisher Scientific, Altrincham, UK) using anti-CD20 BV510 (BioLegend, London, UK), anti-CD3 PE (BioLegend, London, UK), and SYTOX AADvanced Dead Cell Stain (Thermo Fisher Scientific, Altrincham, UK). The percentage of live target cells was calculated relative to tumor cells alone.

### Monocyte-derived macrophage analysis

Monocytes were isolated from PBMCs using the Pan Monocyte Isolation Kit, human (Miltenyi Biotec, Bisley, UK) following the manufacturers’ recommendations and cultured for 7 days in RPMI1640 GlutaMAX (Thermo Fisher Scientific, Altrincham, UK) supplemented with 10% heat-inactivated FBS (TCS Biosciences, Buckingham, UK), 50 μM β-mercaptoethanol (Thermo Fisher Scientific, Altrincham, UK), and 50 ng/mL M-CSF (Thermo Fisher Scientific, Altrincham, UK). Transduction of differentiated macrophages was performed at day 7 by adding appropriate dilutions of vectors in the presence or absence of nevirapine. Cells were maintained for 7 days and then detached using StemPro Accutase Cell Dissociation Reagent (Thermo Fisher Scientific, Altrincham, UK) and pelleted by centrifugation at 300 × *g* for 5 min before DNA extraction performed on a QIAcube HT instrument using the QIAamp 96 DNA QIAcube HT Kit (QIAGEN, Manchester, UK).

### Statistical analysis

Statistical analysis was performed in GraphPad Prism 9 version 9.3.1 for Windows (GraphPad, San Diego, CA, USA) using tests as indicated in the figure legends. *p* < 0.05 was considered significant.

## Data availability

The authors confirm that materials and protocols will be made available on request, subject to appropriate agreements.

## Acknowledgments

The work was funded by Oxford Biomedica (UK) Ltd. We would like to thank other members of the research group at Oxford Biomedica (especially Oliver Pearson, Mat Veal, Daniel Beck, Laura Moyce, Megan Jackson, Anurag Kulkarni, John O’Driscoll, and Andre Raposo) for their contributions to this project. NHS Blood and Transplant have provided material in support of the research. This report is independent research. The views expressed in this publication are those of the author(s) and not necessarily those of NHS Blood and Transplant. Graphical abstract was created in BioRender. Farley, D. (2025) https://BioRender.com/a07g305. The work was performed at Oxford Biomedica (UK) Ltd., Windrush Court, Transport Way, Oxford, Oxfordshire, OX4 6LT, United Kingdom.

## Author contributions

Investigation and methodology, T.C., A.L.K., A.R.B., L.W., D.P., Z.H., J.L., G.D., S.I., E.B., S.F., D.M.J., and B.M.A. Conceptualization and supervision, T.C., A.R.B., J.W., D.C.F., D.M.O., R.M.R., K.A.M., Y.L., and R.N. The manuscript was drafted by T.C. and R.N. and revised by R.N., T.C., K.A.M., Y.L., D.M.O., and D.C.F. All authors read and approved the manuscript.

## Declaration of interests

All authors were, or are, employees of Oxford Biomedica (UK) and received compensation in the form of salary and share options. Some of the work in this manuscript is related to patent applications filed by Oxford Biomedica (UK), including a patent describing the mixed envelope invention (WO 2024/038266 A1, on which T.C. and R.N. are coinventors) and patents describing the TetraVecta system (J.W. and D.C.F. are coinventors on WO2021160993A1, WO2021014157A1, and WO2023062365A2; D.C.F. is an inventor on WO2021094752A1). Y.L. is Director of an independent consulting company (Elexion Consulting) focusing on the cell and gene therapy space.

## Supplemental information

Document S1. Figures S1–S4 and Table S1

Document S2. Article plus supplemental information
