# Supplementary material for: Efficient in vivo generation of CAR T cells using a retargeted fourth-generation lentiviral vector
Source: Mol Ther. 2025 Jul 16;33(10):4953–67. doi: 10.1016/j.ymthe.2025.07.006 (PMC12848154; doi:10.1016/j.ymthe.2025.07.006)
Supplement: Document S1. Figures S1–S4 and Table S1 [file mmc1.pdf]

YMTHE, Volume *33*

Supplemental Information

Efficient *in vivo* generation of CAR T cells using a retargeted fourth-generation lentiviral vector

Tiziana Coradin, Amy L. Keating, Alun R. Barnard, Lynsey Whilding, Diana Pombal, Zara Hannoun, Jack Lewis, Gayathri Devarajan, Sharifah Iqball, Emma Burton, Sara Ferluga, Daniel M. Jones, Ben M. Alberts, Jordan Wright, Daniel C. Farley, Deirdre M. O'Connor, Ravi M. Rao, Kyriacos A. Mitrophanous, Yatish Lad, and Rachael Nimmo

Table S1. Summary of vector and treatment group information for the *in vivo* study.

SupT1 titre refers to functional titre (transducing units (TU)/mL) calculated by qPCR analysis of vector copy number in the SupT1 cell line. Note the CD3^{DARPin}-LV could not be titred on SupT1 cells as this cell line is not permissive for transduction by this vector. Physical titre refers to the number of vector particles per millilitre (vp/mL) calculated by qRT-PCR analysis of RNA copy number. PBMC titre refers to the functional titre (TU/mL) calculated by flow cytometry analysis of CAR expression in PBMCs as a proportion of the target cell population (CD3^{+}, CD8^{+} or all live cells). 200 µL volume was injected per animal. Some vectors were diluted prior to injection. The vector dilution factor, the dose (total TU) administered (calculated using the SupT1 titre) and the number of particles administered per mouse, are indicated for each treatment group. N = Number of mice remaining for analysis in each treatment group at D3 after injection. N/A: Not applicable.

Vector/Group	SupT1 Titre (TU/mL)	Physical Titre (vp/mL)	PBMC titre (TU/mL)	Dilution	Dose (TU)	Dose (Particles)	N
CD3^{DARPin}-LV	N/A	9.9E+11	9.2E+06	N/A	N/A	2.0E+11	9
CD8^{DARPin}-LV	1.8E+08	1.6E+12	1.5E+07	1:2	1.8E+07	1.6E+11	6
CD8^{VHH}-LV	1.8E+08	1.4E+12	4.5E+07	1:2	1.8E+07	1.4E+11	10
VSV-LV (High dose)	1.5E+09	1.4E+11	1.7E+08	N/A	2.9E+08	2.8E+10	12
VSV-LV (Medium dose)	N/A	N/A	N/A	1:8	3.7E+07	3.5E+09	12
VSV-LV (Low dose)	N/A	N/A	N/A	1:16	1.8E+07	1.7E+09	12
Vehicle (TSSM)	N/A	N/A	N/A	N/A	N/A	N/A	11

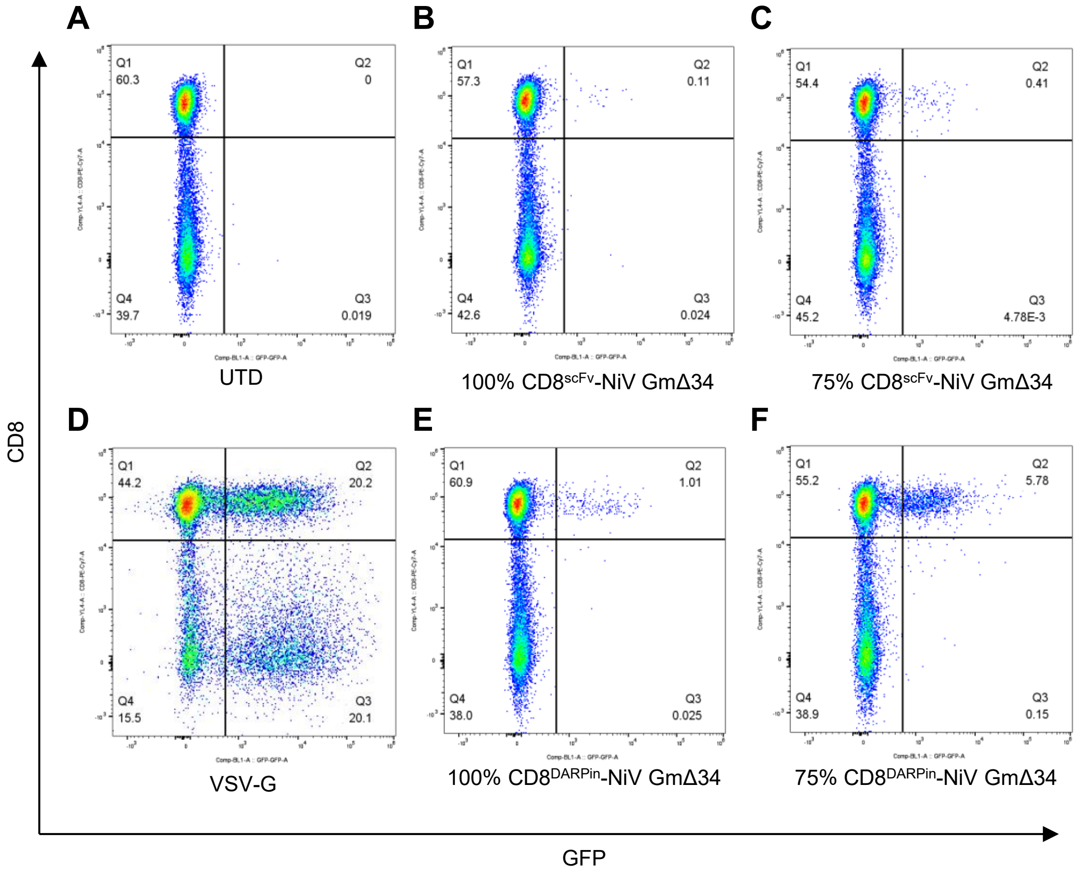

Figure S1. Use of a DARPin and the "mixed" envelope composition improved the transduction efficiency of retargeted LV vectors pseudotyped with a modified NiV envelope (related to Figure 1). Representative flow cytometry plots of data summarised in Figure 1B showing GFP expression in $CD8^+$ T cell populations from PBMCs transduced with retargeted GFP vectors. Vectors were pseudotyped with a retargeted NiV envelope consisting of the NiV FΔ22 fusion protein and retargeted CD8-NiV GmΔ34 attachment protein displaying scFv or DARPin binders for CD8 ($CD8^{scFv}$-NiV and $CD8^{DARPin}$-NiV GmΔ34 respectively), where the G protein component was composed of either 100% retargeted CD8-NiV GmΔ34, or a "mixed" envelope with 75% retargeted CD8-NiV GmΔ34 and 25% non-targeted NiV GmΔ34 proteins. VSV-G pseudotyped vector is shown to compare specificity, but due to the difference in titre, VSV-G vector was diluted 1:100 whereas all retargeted vectors were diluted 1:25. (A) Untransduced (UTD); (B) 100% $CD8^{scFv}$-NiV GmΔ34; (C) 75% $CD8^{scFv}$-NiV GmΔ34; (D) VSV-G; (E) 100% $CD8^{DARPin}$-NiV GmΔ34; (F) 75% $CD8^{DARPin}$-NiV GmΔ34.

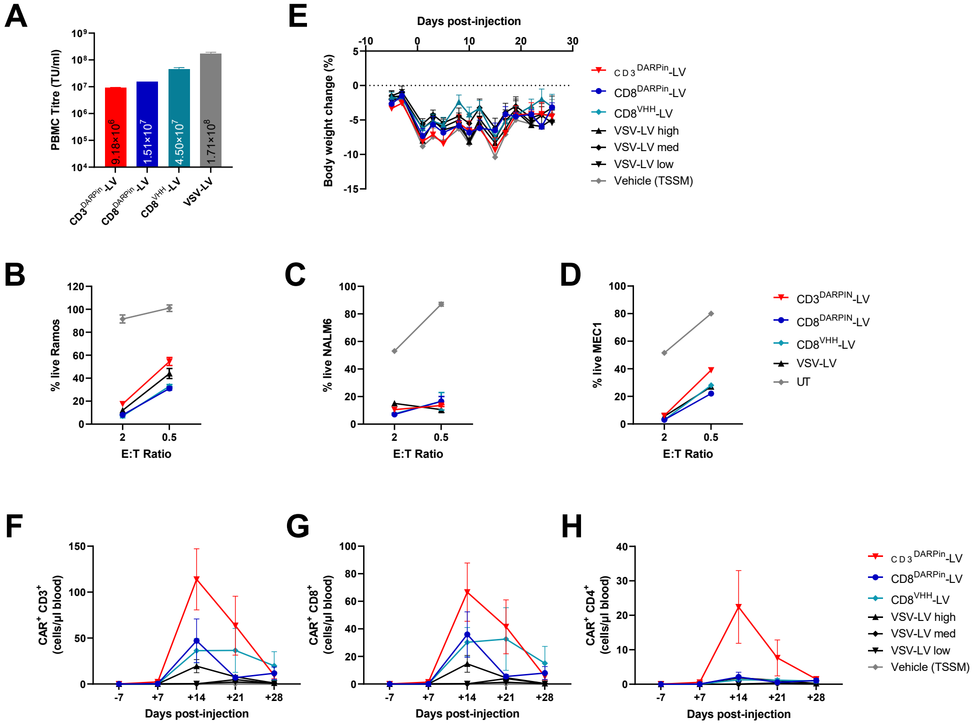

Figure S2. Rapid and efficient generation of CAR T cells *in vivo* by intravenous injection of 4th generation SupA2KO-LV vectors targeted to CD3$^+$ or CD8$^+$ T cells (related to Figure 4). (A) Functionality of the vectors was confirmed by analysing the transduction efficiency on the target population in PBMCs. The proportion of CAR$^+$ cells in the target population, and number of target antigen-expressing cells present at transduction was used to estimate the PBMC titre. Target populations: CD3$^+$ cells for CD3^{DARPin}-LV; CD8+ cells for CD8^{DARPin}-LV and CD8^{VHH}-LV, or all live cells for VSV-LV. (B-D) Cytotoxic activity of the CAR T cells generated by each of the vectors was assessed on multiple CD19$^+$ cell lines (RAMOS, NALM6 and MEC1). (A-D) Data shown as mean ± SD. (E) Longitudinal analysis of body weight of animals injected with vectors or TSSM vehicle control over 4 weeks. (F-H) Absolute numbers of CAR$^+$ T cells per µL of blood over time for different T cell populations: (F) CAR$^+$ CD3$^+$; (G) CAR$^+$ CD8$^+$; (H) CAR$^+$ CD4$^+$. (E-H) Data shown as mean ± SEM (*n* = 6-12 per group).

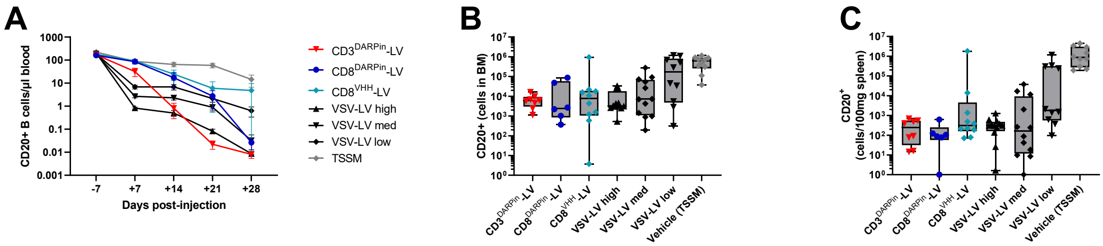

Figure S3. CAR T cells generated *in vivo* using 4th generation retargeted SupA2KO-LV vectors induce rapid and sustained B cell aplasia (related to Figure 5). Absolute B cell numbers corresponding to frequency data shown in Figure 5. (A) Blood analysis: expressed as number of CD20$^+$ cells/µL, mean ± SEM (*n* = 6-12 per group). (B) BM analysis: total CD20$^+$ cells in BM from 4 long bones. (C) Spleen analysis: expressed as the number of cells/100mg. Box and whisker plots with individual data points (*n* = 6-12 per group).

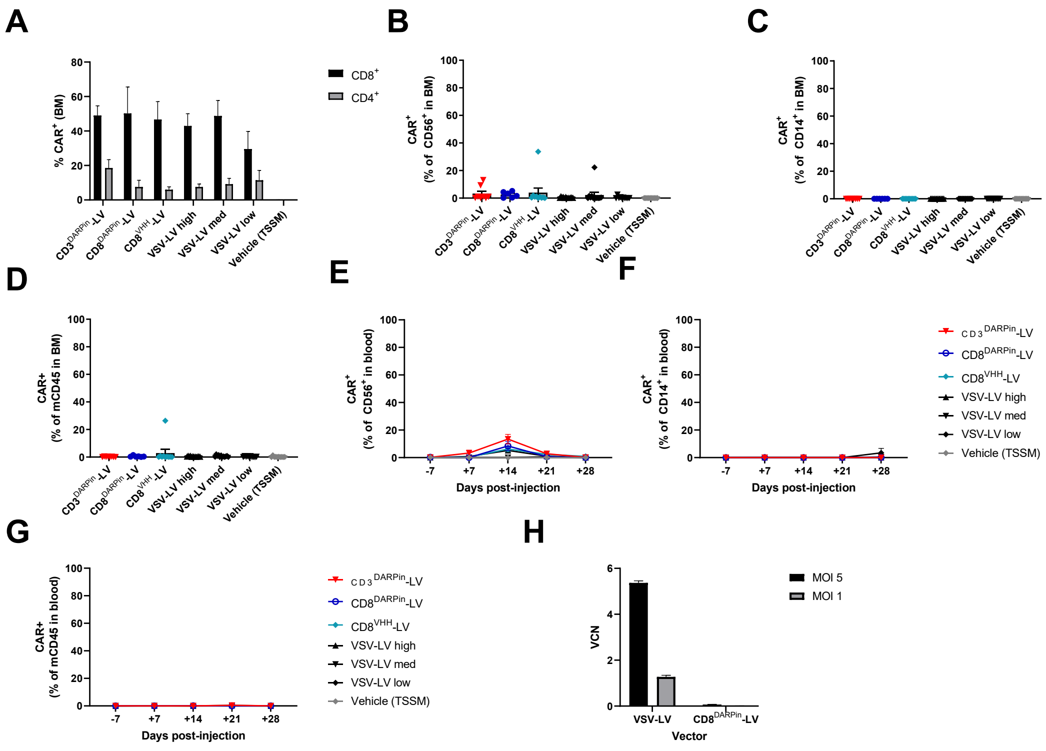

Figure S4. Specific targeting of T cells *in vivo* with 4th generation retargeted SupA2KO-LV vectors (related to Figure 6). (A) Proportion of CAR$^+$ cells within CD8$^+$ and CD4$^+$ T cells in the BM. (B-G) Assessment of CAR expression in other immune cells in the BM (B-D) and blood (E-G). Proportion of CAR$^+$ (B,E) CD56$^+$ NK cells; (C,F) CD14$^+$ monocytes; (D,G) mCD45$^+$ cells. Data shown as mean ± SEM (*n* = 6-12 per group). Individual data points also shown for B-D. (H) Vector copy number per cell assessed by qPCR in monocyte-derived macrophages 7 days after transduction with either VSV-G pseudotyped LV vector or the CD8^{DARPin}-NiV pseudotyped LV vector with the “mixed” envelope at two different MOI (1 and 5). Data shown as mean ± SD (*n*=2).
